# The 2023 Türkiye-Syria earthquakes: analysis of pediatric victims with crush syndrome and acute kidney Injury

**DOI:** 10.1007/s00467-024-06307-7

**Published:** 2024-02-15

**Authors:** Serra Sürmeli Döven, Özlem Tezol, Edanur Yeşil, Fatma Durak, Merve Mısırlıoğlu, Mehmet Alakaya, Feryal Karahan, İsa Kıllı, Mehtap Akça, Semra Erdoğan, Mevlüt Can, Ali Delibaş

**Affiliations:** 1 Faculty of Medicine, Department of Pediatric Nephrology, Mersin University, Mersin, Türkiye; 2 Faculty of Medicine, Department of Pediatrics, Mersin University, Mersin, Türkiye; 3 Faculty of Medicine, Department of Pediatric Infectious Diseases, Mersin University, Mersin, Türkiye; 4 Faculty of Medicine, Department of Pediatric Intensive Care, Mersin University, Mersin, Türkiye; 5 Faculty of Medicine, Department of Pediatric Hematology, Mersin University, Mersin, Türkiye; 6 Faculty of Medicine, Department of Pediatric Surgery, Mersin University, Mersin, Türkiye; 7 Faculty of Medicine, Department of Biostatistics and Medical Informatics, Mersin University, Mersin, Türkiye

**Keywords:** Acute kidney injury, Crush Syndrome, Earthquake, Myoglobin

## Abstract

**Background:**

On February 6th, 2023, two consecutive earthquakes struck southeastern Türkiye with magnitudes of 7.7 and 7.6, respectively. This study aimed to analyze the clinical and laboratory findings, as well as management of pediatric victims with Crush Syndrome (CS) and Acute Kidney Injury (AKI).

**Methods:**

The study included pediatric earthquake victims who were presented to Mersin University Hospital. Clinical and laboratory characteristics of the patients were collected retrospectively.

**Results:**

Among 649 patients, Crush injury (CI), CS and AKI was observed in 157, 59, and 17 patients, respectively. White blood cell count (12,870 [IQR: 9910–18700] vs. 10,545 [IQR: 8355–14057] /µL, P < 0.001), C-reactive protein (51.27 [IQR: 14.80–88.78] vs. 4.59 [1.04–18.25] mg/L, P < 0.001) and myoglobin levels (443.00 [IQR: 198.5–1759.35] vs. 17 [11.8–30.43] ng/ml) were higher in patients with CS, while their sodium (IQR: 134 [131–137] vs. 136 [134–138] mEq/L, P < 0.001) levels were lower compared to non-CS patients. An increase in myoglobin levels was identified as an independent risk factor for developing CS (OR = 1.017 [1.006–1.027]). Intravenous fluid replacement was administered to the patients with CS at a dose of 4000 cc/m^2^/day. Hypokalemia was observed in 51.9% of the CS patients on the third day. All patients with AKI showed improvement and no deaths were reported.

**Conclusions:**

Hyponatremia and increase in inflammation markers associated with CS may be observed. An increase in myoglobin levels was identified as a risk factor for CS. Hypokalemia may be seen as a complication of vigorous fluid therapy during hospitalization.

**Graphical abstract:**

**Figure:**
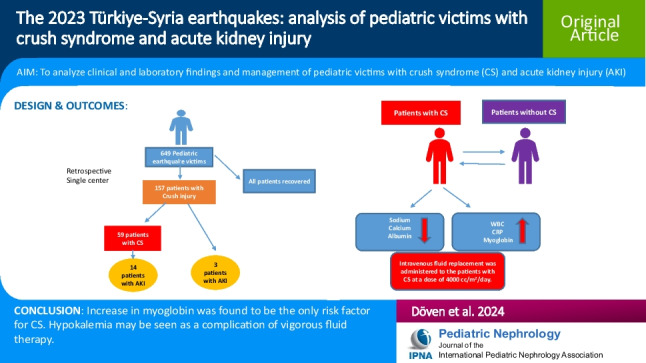
A higher resolution version of the Graphical abstract is available as Supplementary information

**Supplementary Information:**

The online version contains supplementary material available at 10.1007/s00467-024-06307-7

## Introduction

On February 6th, 2023, at 04:17 and 13:24 h, two consecutive earthquakes occurred in southeastern Türkiye, both with the epicenter in Kahramanmaraş with magnitudes of 7.7 and 7.6, respectively. These earthquakes affected a large region of Türkiye, encompassing 11 provinces. Over 50,000 people died, more than 100,000 people were injured and about 150,000 buildings collapsed. This event has been described as the disaster of the century [1].

Earthquakes, commonly seen in Türkiye due to its geographical location, are a major cause of mortality and morbidity. Although they are not preventable, lessons can be learned from earthquakes to promote health care.

Crush injury (CI) is caused by prolonged pressure on the muscle cells. Clinical findings of CI include swelling, erythema, blisters, fractures and tissue necrosis. Crush Syndrome (CS) is the systemic manifestation of rhabdomyolysis due to prolonged continuous pressure on muscle tissue. It may cause hypovolemic shock, acute kidney failure, compartment syndrome, hyperkalemia, hypocalcemia and metabolic acidosis [1]. CS is the second largest cause of death after direct trauma due to the earthquakes.

Although earthquakes have occurred worldwide, a limited number of studies in the literature have addressed the characteristics and management of pediatric victims [2–7]. Therefore, the present study aimed to review the clinical and laboratory characteristics and the management of the pediatric earthquake victims with CS and AKI who were referred and presented to our center.

## Patients and methods

The study included patients who presented to the Pediatric Emergency Service of Mersin University Hospital. Approval was obtained from the Mersin University Ethics Committee (2023/162) to conduct the study. Demographic characteristics and laboratory findings of the patients were extracted from their records. Data were collected by ÖT, EY, FD, MM, MA, FK, İK, MA, MC, AD, while SE conducted the data analysis. SSD designed the study and wrote the manuscript. The study retrospectively collected information about the time spent under the rubble and the time until admission (the time between the date of the earthquakes to the admission to the hospital), type of injury, need for fasciotomy, duration of hospitalization, treatment modalities, fluid-electrolytes administered, the need for fresh/frozen plasma, albumin infusions, dialysis, and prognosis. CI was diagnosed in injured patients based on the presence of compression with swelling in the affected part of the body. CS was diagnosed in patients with CI who exhibited myoglobinuria and/or hematuria, kidney failure, and whose peak creatine kinase (CK) levels were > 1000 U/L [6, 7]. The definition of AKI was made according to urinary output findings (< 0.5 ml/kg/hr, at least for 6–12 h or anuria ≥ 12 h) and serum creatinine values consistent with the KDIGO guideline. Complete blood counts, including hemoglobin (Hgb), white blood cell (WBC) count, platelet (Plt), as well as biochemical analysis, including urea, creatinine, uric acid, sodium, potassium, calcium, phosphorus, alanine transaminase (ALT), aspartate aminotransferase (AST), lactate dehydrogenase (LDH), CK, and C-reactive protein (CRP) of the hospitalized patients were measured. Laboratory parameters were evaluated based on the reference levels of the biochemistry laboratory. Patients with CS and/or AKI were compared to those without CS and/or AKI, in terms of age, time spent under the rubble, time until admission, injured extremity and fracture number, duration of hospitalization, types of injuries and laboratory values. The factors associated with the duration of hospitalization were analyzed.

## Statistical analyses

The Shapiro–Wilk test was used to evaluate normality for continuous measurements. The Student t-test and Mann–Whitney U test were used to compare continuous measurements for normally and non-normally distributed groups, respectively. Descriptive statistics were presented as means, standard deviation, median and 25–75 percentiles. The Pearson chi-square, Fisher’s exact chi-square, and likelihood ratio chi-square tests were used to analyze the differences in the categorical variables. Categorical data were presented as numbers and percentages. The Spearman correlation coefficient was used to determine the correlations between the continuous measurements. Parameters considered to affect CS and AKI were analyzed using logistic regression analysis. A significance level of P < 0.05 was considered statistically significant. For AKI and CS, the Backward Wald method was used in the logistic regression analysis, incorporating significant parameters identified through univariate analysis in the model. The results were presented using tables. For CS, the goodness-of-fit of the model was assessed using the Hosmer–Lemeshow test (χ^2^ = 1.276; p = 0.989), finding that the model showed good fit to the data. In addition, the overall correct classification rate of the model was 87.3%. Concerning the AKI parameter, the model’s fit to the data was assessed using the Hosmer–Lemeshow test (χ^2^ = 13,998; p = 0.082), indicating a good fit to the model. Furthermore, the overall correct classification rate of the model for AKI was 90.0%.

## Results

A total of 649 pediatric earthquake victims presented to our center, of whom 104 were hospitalized. Of these victims, 45.8% were female and 54.2% were male. The mean age of the patients was 92.5 ± 66.6 (1–216) months. Of the patients, 157 (24.9%) had spent time under the rubble. The median amount of time spent under the rubble was 240 [IQR: 60–900] min. The types of injuries are listed in Table 1. Upper, lower, and both upper and lower extremity injuries were present in 34 (7.6%), 32 (7.1%), and 5 (1.1%) patients, respectively. Other complaints of the victims on admission were fever, diarrhea, vomiting, convulsion, laceration, and wounds (n = 492). Oliguria, hematuria, and myoglobinuria were present in 3.9%, 4.3%, and 3.7% of the patients, respectively. Of the patients, 12 were hospitalized in the intensive care unit. The laboratory anomalies of the patients were hyponatremia in 80/281 (28.5%), hyperkalemia in 9/226 (4%), hypokalemia in 25/226 (11.1%), hypocalcemia in 20/136 (14.7%), hyperphosphatemia in 22/135 (16.3%), hyperuricemia in 20/108 (18.5%), increased ALT in 60/189 (31.7%), increased AST in 79/193 (40.9%), increased LDH in 62/109 (56.9%), increased CRP in 145/273 (53.1%), hypoalbuminemia in 26/95 (27.4%), and metabolic acidosis in 16/99 (16.2%) patients. Of the patients, 171 received intravenous fluid [0.9% saline (n = 13), 0.9% saline + 5% dextrose (n = 77), 0.45% saline + 5% dextrose (n = 66), and one-third isotonic saline in 5% dextrose (n = 15)]. In addition, 642 patients were discharged, while 7 patients were referred to other centers. None of the patients died.

**Table 1:** Type of injuries with CS and non-CS patients

Associated injuries	Crush syndrome (n = 59)	Non-crush syndrome (n = 585)	P
Soft tissue	43 (72.9%)	102 (17.4%)	< 0.001
Central nervous system bleeding	18 (30.5%)	25 (4.3%)	< 0.001
Vertebral column	5 (8.5%)	5 (0.9%)	0.001
Peripheral nerve	16 (27.1%)	4 (0.7%)	< 0.001
Thoracic compression	8 (13.8%)	4 (0.7%)	< 0.001
Pneumothorax	5 (8.5%)	2 (0.3%)	< 0.001
Pneumomediastinum	7 (11.7%)	2 (0.3%)	< 0.001
Extremity fractures	17 (28.8%)	13 (2.2%)	< 0.001
Retroperitoneal hematoma	3 (5.1%)	5 (0.9%)	0.029
Pelvic fracture	6 (10.2%)	6 (1.0%)	< 0.001
Costal fracture	4 (6.8%)	4 (0.7%)	0.003
Pulmonary contusion	4 (6.8%)	3 (0.5%)	0.002
Compartment syndrome	16 (27.1%)	1 (0.2%)	< 0.001

## Characteristics of the patients with CS

Of patients with CS (n = 59), 49.2% were female and 50.8% were male. Additionally, 96.6% of the patients with CS were hospitalized, of whom 17% were in the intensive care unit. Compartment syndrome was observed in 28.8% of patients with CS.

Regarding the types of injuries, CS was observed more frequently in patients with all types of injuries (Table 1). Patients with CS were older than non-CS patients. The time spent under the rubble was longer, while the time until admission was shorter in the patients with CS compared to non-CS patients. The number of injured extremities was higher, while there was no difference in the number of fractures and duration of hospitalization in the patients with CS compared to non-CS patients (Table 2).

**Table 2:** Comparison of clinical features of patients with CS and non-CS

Variable		Mean ± SD	P
Age (Months)	CS (n = 59)	124.3 ± 60.4	< 0.001
	Non-CS (n = 585)	88.7 ± 66.2	
Injured extremity number	CS (n = 43)	1.4 ± 0.6	0.021
	Non- CS (n = 43)	1.2 ± 0.4	
		Median [%25–75 percentiles]	P
Time spent under the rubble (min)	CS (n = 52)	720 [255–1620]	< 0.001
	Non- CS (n = 88)	120 [25–480]	
Time until admission (days)	CS (n = 59)	2 [1-3]	< 0.001
	Non- CS (n = 581)	7 [4-10]	
Fracture number	CS (n = 25)	1 [1-2]	0.947
	Non-CS (n = 31)	1 [1-2]	
Hospitalization duration (days)	CS (n = 58)	6.5 [4–13.9]	0.258
	Non-CS (n = 44)	6 [2.25–10.75]	

AKI was observed in 14 (23.7%) patients with CS. Hemodialysis (HD) was performed for seven of them, whereas seven recovered with supportive treatment.

All patients with CS received 0.45% saline + 5% dextrose and 50 mEq/L sodium bicarbonate at a dose of 3000–4000 mL/m^2^/day. Intervention requirements such as fasciotomy, amputation, hyperbaric oxygen, albumin, erythrocyte suspension, fresh/frozen plasma infusions, HD, and intubation were higher in the CS patients compared to the non-CS patients (Table 3).

**Table 3:** Intervention requirements for patients with CS and non-CS

Intervention	Patients with CS (n = 59)	Patients with non-CS (n = 590)	P
Fasciotomy	10	1	< 0.001
Amputation	8	0	
Hyperbaric oxygen	3	0	
Albumin infusions	10	0	
Erythrocyte suspension transfusion	18	7	< 0.001
Fresh/frozen plasma infusions	10	2	< 0.001
Hemodialysis	7	3	< 0.001
Intubation	7	1	< 0.001

The patients with CS had higher WBC count, CK, myoglobin, urea, uric acid, ALT, AST, LDH, and CRP levels, while they had lower sodium, albumin, and calcium levels compared to non-CS patients. There was no statistical difference in the Hgb, Plt, potassium, phosphorus and creatinine levels between the CS and non-CS patients (Supplementary Table 1).

The only risk factor for CS was myoglobin values according to logistic regression analysis. Accordingly, as myoglobin values increase, the risk of CS increases by 1.017 times (p = 0.002) (Table 4).

**Table 4:** Logistic regression analysis for CS

	B	Wald	OR [% 95 CI]	P
Age	-0.006	0.501	0.994 [0.977–1.011]	0.479
Time until admission	-0.237	0.598	0.789 [0.433–1.438]	0.439
Myoglobin	0.016	9.306	1.017 [1.006–1.027]	**0.002**
Urea	-0.097	2.095	0.908 [0.797–1.035]	0.148

The laboratory findings of the patients with CS on the first and third days of hospitalization, respectively, were as follows: an increase in creatinine levels in 11/58 (19%) and 7/55 (12.7%); hyponatremia in 28/57 (49.1%) and 12/55 (21.8%); hyperkalemia in 7/59 (11.9%) and 0.00%; hypokalemia in 6/59 (10.2%) and 28/54 (51.9%); hyperuricemia in 19/59 (32.2%) and 8/48 (16.7%); hypocalcemia in 16/55 (29.1%) and 26/54 (48.1%); hyperphosphatemia in 14/57 (24.6%) and 11/50 (22%); increase in uric acid in 13/49 (26.5%) and 2/35 (5.7%); increased ALT in 47/59 (79.7%) and 39/54 (72.2%); increased AST in 52/59 (88.1%) and 40/53 (75.5%); increased LDH in 43/44 (97.7%) and 26/32 (81.3%); increased CRP in 49/53 (92.5%) and 36/44 (81.8%); hypoalbuminemia in 17/27 (63%) and 33/40 (82.5%); and metabolic acidosis in 10/42 (23.8%) and 0.00%.

## Characteristics of the patients with AKI

AKI was observed in 17/649 (2.6%) patients. No statistical difference was found between the males and females in terms of AKI (P = 0.146). CS was observed more in patients with AKI compared to non-AKI patients (82.4% vs. 10.1%, P < 0.001) The patients with AKI were older than non-AKI patients. The time spent under the rubble was longer while the time until admission was shorter in patients with AKI compared to non-AKI patients. There was no statistical difference in terms of injured extremity number, fracture number and duration of hospitalization between AKI and non-AKI patients (Table 5).

**Table 5:** Comparison of clinical features of patients with AKI and non-AKI

Variable		Mean ± SD	P
Age (Months)	AKI (n = 17)	130.4 ± 62.3	0.019
	Non-AKI (n = 437)	91.6 ± 66.7	
Injured extremity number	AKI (n = 11)	1.6 ± 0.7	0.118
	Non-AKI (n = 68)	1.3 ± 0.5	
		Median [% 25–75 percentiles]	
Time spent under the rubble (min)	AKI (n = 15)	480 [312–960]	0.041
	Non-AKI (n = 100)	240 [30–864]	
Time until admission (days)	AKI (n = 17)	3 [1.5–3.0]	< 0.001
	Non-AKI (n = 437)	7 [3-10]	
Fracture number	AKI (n = 6)	1 [1-2]	0.429
	Non-AKI (n = 43)	1 [1-2]	
Hospitalization duration (days)	AKI (n = 17)	7 [4-13]	0.605
	Non-AKI (n = 77)	6 [3-13]	

HD was performed in 10 patients with AKI. All patients’ kidney functions showed improvement after HD. Continuous kidney replacement therapy was performed in one patient. The median number of HD sessions was 2 [IQR: 1.75–5.25]. The remaining six patients recovered with only supportive treatment.

The patients with AKI had higher WBC count, CK, myoglobin, creatinine, ALT, AST, LDH, CRP, urea, phosphorus and uric acid levels, while they had lower sodium and albumin levels compared to non-AKI patients (Supplementary Table 2).

The only risk factor for AKI was creatinine value according to logistic regression analysis. Accordingly, as creatinine values increased, the risk of AKI increased by 5.469 times (p = 0.035) (Table 6).

**Table 6:** Logistic regression analysis for AKI

	B	Wald	OR [% 95 CI]	P
Age	-0.011	1.697	0.989 [0.973–1.006]	0.193
Creatinine	1.699	4.437	5.469 [1.125–26.577]	**0.035**
AST	-0.006	2.229	0.994 [0.986–1.002]	0.135
LDH	0.003	3.207	1.003 [1.000-1.006]	0.073

AST, aspartate aminotransferase; LDH, lactate dehydrogenase

## Factors associated with the duration of hospitalization

The mean duration of hospitalization was 9.4 ± 10.0 (1–62) days (n = 104). The duration of hospitalization was positively correlated with the admission levels of WBC, CK, myoglobin, urea, potassium, uric acid, ALT, AST, LDH, and CRP. It was negatively correlated with calcium levels. Age, time spent under the rubble, the number of injured extremities and fractures, the number of HD sessions, and admission levels of Hgb, Plt, creatinine, sodium, albumin and phosphorus were not associated with the duration of hospitalization (Table 7).

**Table 7:** Factors associated with the duration of hospitalization

Variables	r	p
Age (months)	0.043	0.664
Spent time under the rubble	0.183	0.111
Number of injured extremities	0.183	0.173
Number of fractures	0.328	0.051
Number of hemodialysis sessions	0.553	0.098
Hgb (g/dl)	0.034	0.736
WBC (/µL)	0.280	0.004
Plt (/µL)	-0.034	0.734
CK (U/L)	0.320	0.004
Myoglobin (ng/ml)	0.384	< 0.001
Urea (mg/dl)	0.265	0.007
Creatinine (mg/dl)	0.170	0.867
Sodium (mEq/L)	-0.017	0.867
Potassium (mEq/L)	0.312	0.002
Calcium (mg/dl)	-0.258	0.017
Albumin	-0,167	0.279
Phosphorus (mg/dl)	0.150	0.171
Uric acid (mg/dl)	0.384	0.001
ALT (U/L)	0.218	0.029
AST (U/L)	0.251	0.011
LDH (U/L)	0.349	0.004
CRP (mg/L)	0.207	0.048

Hgb, hemoglobin; WBC, white blood cell count; Plt, platelets; CK, creatinine kinase; ALT, alanine transaminase; AST, aspartate aminotransferase; LDH, lactate dehydrogenase; CRP, C reactive protein

## Discussion

Although earthquakes have occurred worldwide, reports about clinical and laboratory findings, as well as the management of pediatric victims are scarce. The scarcity of these reports can cause certain challenges in collecting data in the chaotic circumstances following earthquakes and the heavy workload that healthcare providers face during those days. In addition to patient management, an evaluation was conducted on the laboratory changes during the hospitalization of patients with CS and factors associated with the duration of hospitalization, which had not been assessed before. These findings may help healthcare providers to manage patients after earthquakes, which are not preventable.

CS is a major cause of death due to natural disasters like earthquakes. CI emerges because of continuous and prolonged pressure on the limbs, whereas CS develops if other organ dysfunction and systemic involvement presents in addition to muscle injury. In a study evaluating Marmara earthquake victims, CS was observed more often in patients between 20 and 59 years of age. Only 1.9% of patients were younger than 10 years [3]. According to our study, the patients with CS were older than 10 years of age, aligning with this finding. This can be attributed to the larger muscle mass and is more often faced with rhabdomyolysis at older ages (10–18 years). At least one hour spent under the rubble is necessary to develop CS [8]. However, according to the aforementioned study, the development of CS was not associated with the time spent under the rubble [3]. According to the study by İskit et al., there was no correlation between the development of CS and age, time spent under the rubble, or the time of evacuation from the disaster area to the hospital [2]. In our study, the time spent under the rubble was longer (median 12 h) in the CS patients compared to the non-CS patients (median 2 h) and the time before admission was shorter compared to non-CS patients. Due to the serious condition of patients with CS, they might have been transferred to the hospital faster, while some of the non-CS patients were admitted to the hospital after arriving by their own means.

The number of injured extremities and all types of injuries were observed more in the patients with CS compared to non-CS patients, as was expected. Clinical findings of CS occur due to rhabdomyolysis. The permeability of muscle sarcolemma increases under pressure [9], leading to the transition of sodium chloride and calcium into the cells, whereas potassium, purine, phosphate, lactic acid, myoglobin, and CK are released from the cells [10]. Since the sarcoplasm is more hypertonic than the extracellular area, water passes into the cells, leading to compartment syndrome and hypovolemia [11, 12], which contributes to the risk of developing AKI. An increase in serum muscle enzyme CK is the prominent laboratory change observed in CS and values between 500 and 3000 U/L are proposed as indicators of rhabdomyolysis [7, 13, 14]. The CK levels observed in patients with CS were very high (median 11,017.5 U/L), which aligned with severity of rhabdomyolysis in our study. Although research has demonstrated that hyperkalemia is a common finding in earthquake victims [15], it was observed in only 4% of all patients in our study and in 11.9% of CS patients. No statistical difference was observed in terms of the potassium levels between CS and non-CS patients.

Among the hospitalized patients with CS, hypokalemia at admission was observed in 6/59 (10.2%) and on the third day, hypokalemia was observed in 28/54 (51.9%) of patients, who were in need of potassium supplementation. These notable changes in potassium level can be attributed to providing vigorous fluid therapy to patients with CS. Both hypo- and hyperalbuminemia can be observed during disasters. Hyperalbuminemia can result from dehydration, while hypoalbuminemia can be related to malnutrition, inflammation, capillary leakage, and fluid overload [16]. In the present study, the albumin levels were significantly lower in the patients with CS compared to non-CS patients. This can be attributed to inflammation and capillary leakage. Other inflammation markers, such as the WBC and CRP levels, were higher in the CS patients. Rhabdomyolysis and infectious complications may also lead to leukocytosis [17, 18].

Anemia and thrombocytopenia are other reported anomalies observed following disasters. Anemia may result from blood loss or hemodilution if the patient receives a large amount of fluid [5]. Thrombocytopenia can be related to disseminated intravascular coagulation [17]. No statistical difference was found between CS and non-CS patients regarding the Hgb and Plt counts. An increase in the ALT, AST, and LDH levels can be observed in patients with CS, aligning with the present study [6]. Hypocalcemia may arise from the transition of calcium from the injured muscle cells, binding with phosphates as calcium-phosphate crystals and from a decrease in the sensitivity to parathormone [12]. İskit et al. [2] found hypocalcemia in patients with CS, as was the case in our patients. The sodium levels were lower in the patients with CS in our study. Although hyponatremia is not a common reported finding in patients with CS, it can be related to the transition of sodium to the intracellular space in patients with CS.

Myoglobin is an important substance that increases in rhabdomyolysis due to release from the injured muscle cells. It was reported that myoglobin serum levels could be found within normal range due to its short half-life [10]. In the present study, myoglobin levels were significantly higher in patients with CS. This could be attributed to rapid presentation of the patients with CS. An increase in myoglobin levels was an independent risk factor for CS which had not been assessed before.

AKI after CS occurs due to hypovolemia, vasoconstriction, and rhabdomyolysis [19]. According to a study by İskit, AKI was observed in 10 out of 15 (66.7%) patients with CI [2]. No correlation was observed between the number of injured extremities, the time spent under the rubble, and AKI. In our study, AKI occurred in 14/59 (23.7%) patients with CS. The time spent under the rubble was longer while there was no significant difference in the injured extremity number in patients with AKI compared to non-AKI patients. According to Sever et al.'s study, 12.0% of the patients had impaired kidney function, of whom 8.9% were treated with dialysis [5]. In our study, AKI was observed in 17/649 (2.5%) patients, of whom 10/649 (1.5%) were treated with dialysis. The rate of AKI in both the CS patients and all patients was lower in our study compared to other studies. This can be related to rapid admission to the hospital from the earthquake area or they might have received fluid therapy before admission to our center. The patients with AKI were older since the patients with CS were older compared to non-CS patients.

All patients with CS received 0.45% saline + 5% dextrose and 50 mEq/L sodium bicarbonate at a dose of 3000–4000 mL/m^2^/day, as recommended in Sever’s study [10]. It was reported that complications of CS can be prevented by early and vigorous fluid replacement treatment [4]. Vigorous fluid replacement therapy may cause hypervolemia, hypertension, heart failure and pulmonary edema in patients with oliguria. Thus, the amount of the fluid given to patients should be adjusted based on their physical examinations and urine outputs.

Fasciotomy and amputation were performed on 16.07% and 13.6% of patients with CS, respectively. According to a study by Demirkıran et al. [4], fasciotomy and amputation were performed on 38.9% and 33.3% of patients with CS, respectively. This might have been related to the severity of the patients with CS in their study.

The factors related to the duration of hospitalization were admission levels of WBC, CK, myoglobin, potassium, uric acid, ALT, AST, LDH and CRP – all were positively correlated with the duration of hospitalization. These findings might have been related to the consequences of CS. Regarding the prognoses of the patients, 99% were discharged and none died. All patients with AKI recovered. This might have been related to the rapid admission of the patients to the hospital, early fluid replacement, and close follow-up of the patients with CS, who had a high risk of mortality.

The limitation of the current study is its retrospective design. No information could be collected regarding patients receiving therapy before admission. In addition, it was unknown if they were referred or admitted by their own means to our center after the earthquakes. The relatively small sample size with outcomes of CI, CS, AKI, or the need for dialysis was another limitation.

In conclusion, CS after earthquakes is an important cause of mortality and morbidity. Electrolyte and mineral imbalances, in addition to an increase in inflammatory markers, may be observed in association with CS, which requires close follow-up. Increase in myoglobin levels is found to be a risk factor for CS. Hypokalemia may arise as a complication of vigorous fluid therapy during hospitalization.

### Supplementary Information

Below is the link to the electronic supplementary material.

Graphical Abstract (PPTX 77.6 KB)

Supplementary file2 (DOCX 17 KB)

Supplementary file3 (DOCX 18 KB)

## Data Availability

All data generated or analysed during this study are included in this published article and its supplementary information files.
