# Supplementary material for: The 2023 Türkiye-Syria earthquakes: analysis of pediatric victims with crush syndrome and acute kidney Injury
Source: Pediatr Nephrol. 2024 Feb 15;39(7):2209–15. doi: 10.1007/s00467-024-06307-7 (PMC11147823; doi:10.1007/s00467-024-06307-7)
Supplement: Supplementary file 1 — Graphical Abstract (PPTX 77.6 KB) [file 467_2024_6307_MOESM1_ESM.pptx]

## Slide 1
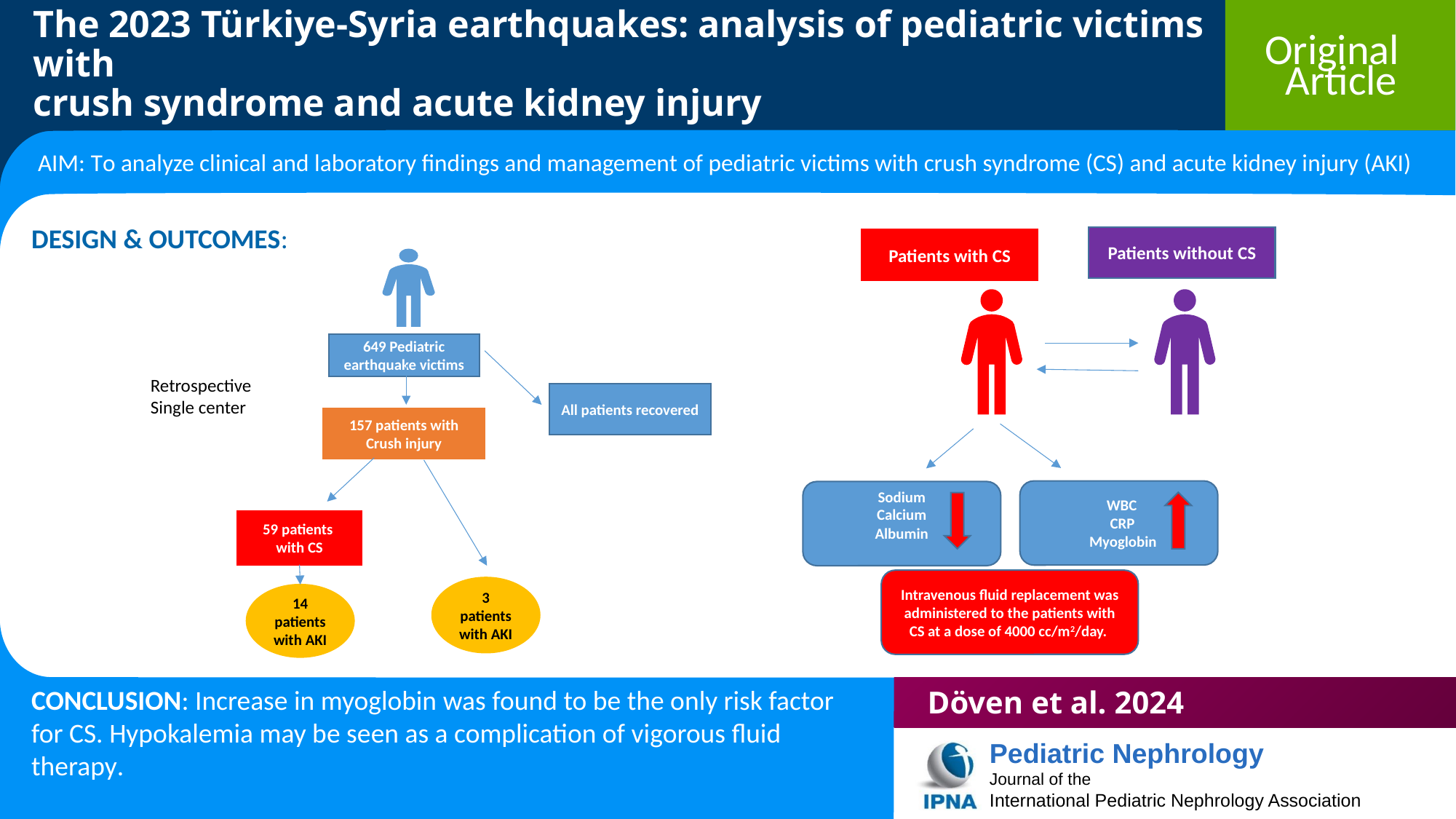

The 2023 Türkiye-Syria earthquakes: analysis of pediatric victims with
crush syndrome and acute kidney injury
AIM: To analyze clinical and laboratory findings and management of pediatric victims with crush syndrome (CS) and acute kidney injury (AKI)
DESIGN & OUTCOMES:
Patients without CS
Patients with CS
649 Pediatric earthquake victims
Retrospective
Single center
All patients recovered
157 patients with Crush injury
 WBC
 CRP
Myoglobin
Sodium
Calcium
Albumin
59 patients
with CS
Intravenous fluid replacement was administered to the patients with CS at a dose of 4000 cc/m2/day.
3 patients with AKI
14 patients with AKI
CONCLUSION: Increase in myoglobin was found to be the only risk factor for CS. Hypokalemia may be seen as a complication of vigorous fluid therapy.
Döven et al. 2024
