# Supplementary material for: The 2023 Türkiye-Syria earthquakes: analysis of pediatric victims with crush syndrome and acute kidney Injury
Source: Pediatr Nephrol. 2024 Feb 15;39(7):2209–15. doi: 10.1007/s00467-024-06307-7 (PMC11147823; doi:10.1007/s00467-024-06307-7)
Supplement: Supplementary file 2 — Supplementary file2 (DOCX 17 KB) [file 467_2024_6307_MOESM2_ESM.docx]

**Supplementary Table 1. Comparison of laboratory levels of patients with CS and non-CS**

| **Mean of laboratory parameters** |  | **Mean ±SD** | **P** |
| --- | --- | --- | --- |
| Hgb levels (g/dl) | CS (n=59) | 11.74±2.90 | 0.854 |
|  | Non-CS (n=227) | 11.81±1.93 |  |
| Plt (µL) | CS (n=59) | 322423.72±141172.45 | 0.554 |
|  | Non-CS (n=227) | 333955.94±131068.98 |  |
| Urea (mg/dl) | CS (n=59) | 41.08±35.92 | 0.009 |
|  | Non-CS (n=222) | 27.96±20.38 |  |
| Potassium (mEq/L) | CS (n=59) | 4.23±0.915 | 0.094 |
|  | Non-CS (n=163) | 4.01±0.51 |  |
| Albumin (g/L) | CS (n=26) | 31.55±5.29 | **<0.001** |
|  | Non-CS (n=67) | 40.16±5.30 |  |
| Calcium (mg/dl) | CS (n=56) | 8.92±0.81 | **<0.001** |
|  | Non-CS (n=78) | 9.66±0.71 |  |
| Phosphorus (mg/dl) | CS (n=57) | 4.60±2.09 | 0.834 |
|  | Non-CS (n=75) | 4.54±0.92 |  |
| Uric acid (mg/dl) | CS (n=49) | 5.65±3.39 | **0.004** |
|  | Non-CS (n=57) | 4.03±1.98 |  |
|  |  | **Median [%25-75 percentiles]** | **P** |
| WBC (µL) | CS (n=59) | 12870 [9910-18700] | **<0.001** |
|  | Non-CS (n=228) | 10545 [8355-14057] |  |
| CK (U/L) | CS (n=56) | 11017.5 [3296-36982.75] | **<0.001** |
|  | Non-CS (n=98) | 146 [94.25-282] |  |
| Myoglobin (ng/ml) | CS (n=53) | 443.00 [198.5-1759.35] | **<0.001** |
|  | Non-CS (n=55) | 17 [11.8-30.43] |  |
| Creatinine (mg/dl) | CS (n=59) | 0.38 [0.28-0.51] | 0.087 |
|  | Non-CS (n=223) | 0.34 [0.24-0.50] |  |
| Sodium (mEq/L) | CS (n=57) | 134 [131-137] | **<0.001** |
|  | Non-CS (n=220) | 136 [134-138] |  |
| ALT (U/L) | CS (n=59) | 109 [50-239] | **<0.001** |
|  | Non-CS (n=127) | 17 [12-25] |  |
| AST (U/L) | CS (n=59) | 182 [86-471] | **<0.001** |
|  | Non-CS (n=130) | 27.5 [21.82-44.00] |  |
| LD (U/L) | CS (n=43) | 746 [469-1496] | **<0.001** |
|  | Non-CS (n=62) | 244.5 [196.5-285.5] |  |
| CRP (mg/L) | CS (n=53) | 51.27 [14.80-88.78] | **<0.001** |
|  | Non-CS (n=216) | 4.59 [1.04-18.25] |  |

Hgb, hemoglobin; WBC, white blood cell count; Plt, platelets; CK, creatinine kinase; ALT, alanine transaminase; AST,aAspartate aminotransferase; LD, lactate dehydrogenase; CRP, C reactive protein
