# Supplementary material for: The 2023 Türkiye-Syria earthquakes: analysis of pediatric victims with crush syndrome and acute kidney Injury
Source: Pediatr Nephrol. 2024 Feb 15;39(7):2209–15. doi: 10.1007/s00467-024-06307-7 (PMC11147823; doi:10.1007/s00467-024-06307-7)
Supplement: Supplementary file 3 — Supplementary file3 (DOCX 18 KB) [file 467_2024_6307_MOESM3_ESM.docx]

**Supplementary Table 2. Comparison of laboratory levels of patients with AKI and non-AKI**

|  |  | **Mean ±SD** | **P** |
| --- | --- | --- | --- |
| Hgb levels (g/dl) | AKI (n=17) | 12.85 ± 3.77 | 0.229 |
|  | Non-AKI (n=208) | 11.70 ± 2.1 |  |
| Plt (x10^3^ µL) | AKI (n=17) | 273588.24 ± 93848.99 | 0.089 |
|  | Non-AKI (n=208) | 331086.54 ± 136230.35 |  |
| Urea (mg/dl) | AKI (n=17) | 74.18 ± 73.34 | **0.021** |
|  | Non-AKI (n=202) | 28.78 ± 15.16 |  |
| Potassium (mEq/L) | AKI (n=17) | 4.40 ± 1.15 | 0.285 |
|  | Non-AKI (n=167) | 4.09 ± 0.59 |  |
| Albumin (g/L) | AKI (n=10) | 30.91 ± 5.45 | **0.001** |
|  | Non-AKI (n=67) | 38.27 ± 6.37 |  |
| Calcium (mg/dl) | AKI (n=17) | 8.94 ± 1.06 | 0.051 |
|  | Non-AKI(n=106) | 9.38 ± 0.81 |  |
| Phosphorus (mg/dl) | AKI (n=17) | 5.54 ± 2.20 | **0.008** |
|  | Non-AKI (n=104) | 4.44 ± 1.42 |  |
| Uric acid (mg/dl) | AKI (n=13) | 7.87 ± 5.52 | **0.043** |
|  | Non-AKI (n=84) | 4.40 ± 1.98 |  |
|  |  | **Median [%25-75 percentiles]** | P |
| WBC (x10^3^/µL) | AKI (n=17) | 15250 [10835-19770] | **0.020** |
|  | Non-AKI (n=208) | 10905 [8427.5-15365] |  |
| CK (U/L) | AKI (n=15) | 37184 [6716-95438] | **<0.001** |
|  | Non-AKI (n=114) | 322.5 [136.5- 4329.75] |  |
| Myoglobin (ng/ml) | AKI (n=16) | 1606.85 [249.05-4007.00] | **<0.001** |
|  | Non-AKI(n=83) | 61 [17-359] |  |
| Creatinine (mg/dl) | AKI (n=17) | 0.48 [0.35-3.76] | **0.001** |
|  | Non-AKI (n=203) | 0.34 [0.24-0.50] |  |
| Sodium (mEq/L) | AKI (n=17) | 132 [131-137] | **0.020** |
|  | Non-AKI (n=201) | 136 [134-138] |  |
| ALT (U/L) | AKI (n=17) | 175 [54.5-340.5] | **<0.001** |
|  | Non-AKI (n=146) | 22 [15-52.75] |  |
| AST (U/L) | AKI (n=17) | 450 [84-972] | **<0.001** |
|  | Non-AKI (n=148) | 39.5 [25.0-75.8] |  |
| LD (U/L) | AKI (n=14) | 1032 [449.8-2506.8] | **<0.001** |
|  | Non-AKI (n=80) | 302.5 [240.25-548.25] |  |
| CRP (mg/L) | AKI (n=14) | 43.6 [9.9-135.5] | **0.011** |
|  | Non-AKI (n=195) | 8.4 [2.6-40.0] |  |

Hgb, hemoglobin; WBC, white blood cell count; Plt, platelets; CK, creatinine kinase; ALT, alanine transaminase; AST,aAspartate aminotransferase; LD, lactate dehydrogenase; CRP, C reactive protein
